# Society Meetings

**Published:** 1897-06

**Authors:** 


### Society Meetings.

The Buffalo Academy of Medicine held meetings during the month of May as follows:

*Section on Surgery.*—Tuesday evening, May 4th, program: The diagnosis of tumors, by Dr. Chas. E. Congdon; A study of absorption of ligatures, with microscopic demonstration, by Dr. L. A. Hendee; Sudden loss of sight, by Dr. J. C. Clemesha. Exhibition of cases and specimens: Dr. H. E. Hayd, A case of appendicitis, with specimens; Dr. Grover W. Wende, A case of mycosis fungoides; Dr. E. A. Smith, Cases and specimens; Dr. C. P. Smith, A case.

*Section on Medicine.*—Tuesday evening, May 11th, program: Pancreatic disease and fat necrosis, by Dr. H. U. Williams.

*Section on Pathology.*—Tuesday evening, May 18th, program: Brain of a suicide, with fissural peculiarities, by Prof. Burt Green Wilder, of Cornell University. Exhibition of specimens: Mycosis fungoides, by Dr. Grover W. Wende; Gallstone, and the like, by Dr. A. L. Benedict.

*Section on Obstetrics and Gynecology.*—Tuesday evening, May 25th, program: Infantile dyspepsia, its prophylaxis, results and treatment, by Dr. A. L. Benedict; Acetanilid poisoning in a newly born child, absorption from umbilical wound, by Dr. Irving M. Snow.

---

The Medical Society of the County of Cattaraugus held its annual meeting at Salamanca, on Tuesday, May 11, 1897. Officers for the ensuing year were elected as follows: President, Dr. M. C. Hawley, of Randolph; vice-president, Dr. A. W. Smallman, of Ellicottville; secretary and treasurer, Dr. T. B. Laughlen, of Olean; committee on program, Drs. Smallman, Laughlen and Beals; censors, Drs. Bedient, of Little Valley; Lattin, of Cattaraugus and Beals, of Salamanca. An address upon Valvular diseases of the heart was given by Dr. John H. Pryor, of Buffalo, and a paper on Complications of valvular heart diseases was read by Dr. A. D. Lake, of Gowanda. The next meeting of the society will be held in August.

---

The French Association of Urologists is the name of a new society that has been recently organised at Paris, with the object of studying the histology and pathology of the urinary passages. The purpose of this association is to reunite and group those investigators whose works have especially contributed to the progress of this branch of medical science.

Foreign corresponding members will be elected on a vote of two-thirds of the home membership, and are exempt from the payment of the initiation fee and annual dues. Applications should be made to secretary-general, M. Desnos, 31 rue de Rome, Paris.

---

The American Gastro-enterological Association will hold its first meeting at Philadelphia, Thursday, June 3, 1897, at the College of Physicians, n. e. cor. 13th and Locust streets. This is the latest attempt at subdividing the various organs of the body into separate fields for occupation by national special societies. A comprehensive program has been issued and an interesting meeting is predicted.

---

The Medical Society of the County of Erie will hold its regular semi-annual meeting on Tuesday, June 8, 1897, at 10 o'clock A. M., in the rooms of the Buffalo Academy of Medicine. The program is preparing by the secretary, Dr. F. C. Gram.

